# Utility of Iodine-131 hybrid SPECT-CT fusion imaging before high-dose radioiodine therapy in papillary thyroid carcinoma

**DOI:** 10.4103/0972-3919.63599

**Published:** 2010

**Authors:** Anish Bhattacharya, Sunil Hejjaji Venkataramarao, Chandra Sekhar Bal, Bhagwant Rai Mittal

**Affiliations:** Department of Nuclear Medicine, PGIMER, Chandigarh, India; 1 Department of Nuclear Medicine, AIIMS, New Delhi, India

**Keywords:** Carcinoma, hybrid imaging, iodine-131, SPECT-CT, thyroid

## Abstract

The management protocol for differentiated thyroid cancer includes whole body iodine-131 imaging, to detect residual thyroid tissue and distant metastasis, after thyroidectomy. However, the diagnostic dose of radioiodine may fail to detect the non-functioning or poorly functioning metastasis. We present a case where hybrid single photon-emission computed tomographic and computed tomographic (SPECT-CT) fusion imaging, using a diagnostic dose of iodine-131, was able to detect both functioning as well as non-functioning pulmonary metastases, prior to high-dose radioiodine therapy.

## INTRODUCTION

The management protocol for differentiated thyroid cancer includes whole body iodine-131 imaging to detect residual thyroid tissue and distant metastasis after thyroidectomy. However, the diagnostic dose of radioiodine may fail to detect the non-functioning or poorly functioning metastasis. Hybrid SPECT-CT imaging may be used in such cases to detect both the functioning and non-functioning metastases in the same study.

## CASE REPORT

**Figure 1:**
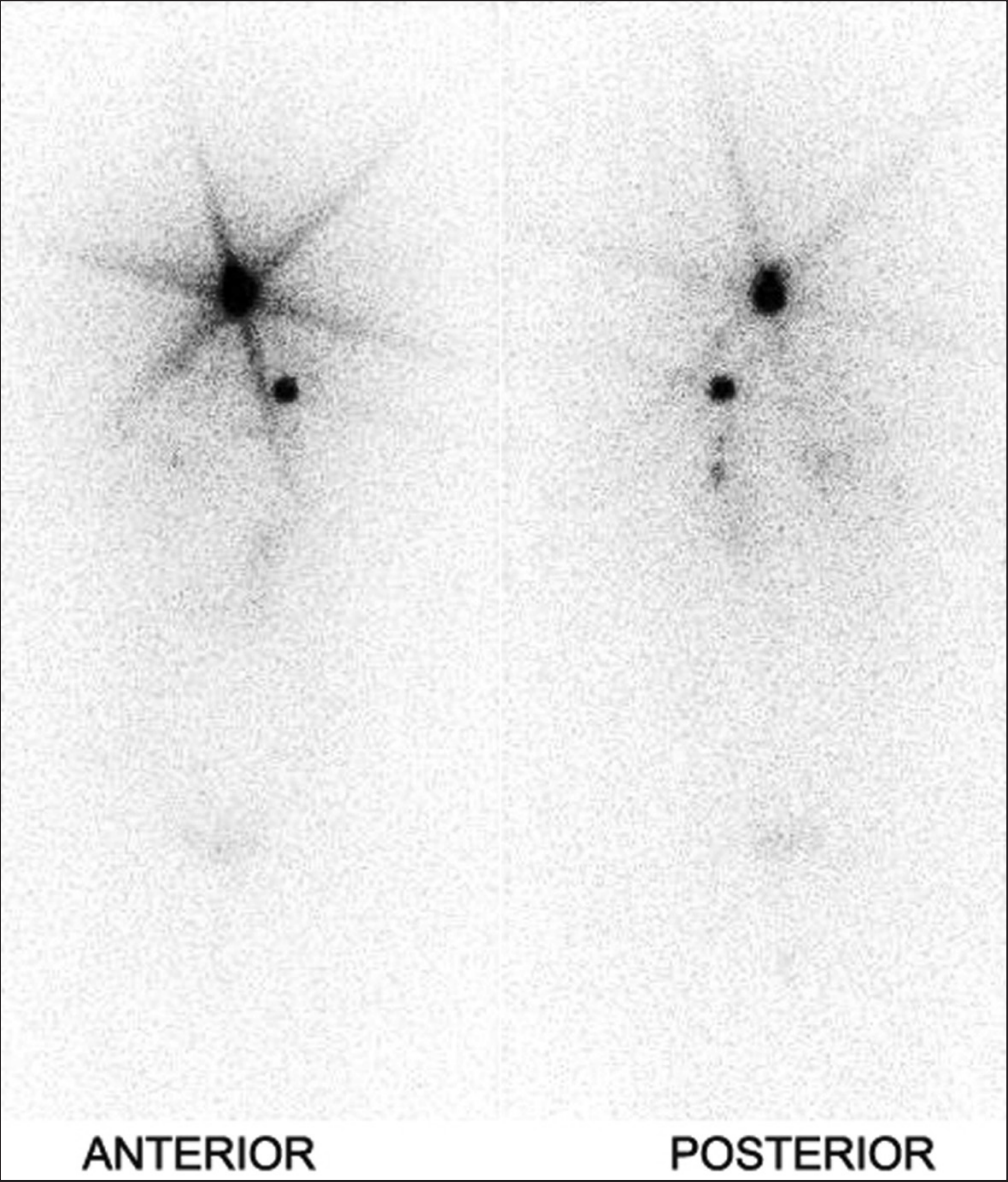
Post-thyroidectomy whole-body Iodine-131 scan showing radioiodine–avid foci in the neck and upper part of the left lung. Mild, irregular tracer uptake is also seen in both lung bases

A 43-year-old Asian female reported to our institute with a mass in the anterior part of her neck, increasing in size for the past one year, with retrosternal extension. Fine needle aspiration cytology was inconclusive with regard to the benign or malignant nature of the mass. The patient underwent total thyroidectomy and papillary thyroid carcinoma was diagnosed on histopathology. Whole body iodine-131 scan (I-131 WBS) was performed after six weeks of thyroxine abstinence, using a dual-head gamma camera fitted with medium energy collimators (Infinia Hawkeye 4, GE, Milwaukee, USA), 48 hours after oral administration of 74 MBq of I-131. A large radioiodine–avid focus was seen in the neck, in the midline, with an additional focus in the upper part of the left lung [Figure 1]. Mild, irregular tracer uptake was also detected in both lung bases, suggestive of pulmonary metastases.

**Figure 2:**
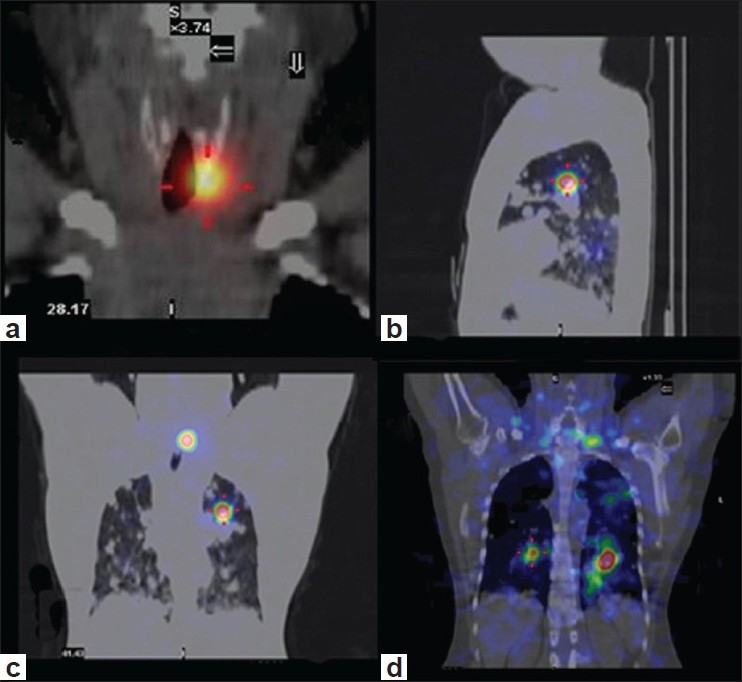
a) Hybrid SPECT-CT fusion shows I-131 uptake in the residual thyroid tissue on the left side of the neck; b-d) Multiple radio-opaque nodules are seen in both lungs, with radioiodine concentration in a few of these nodules

**Figure 3:**
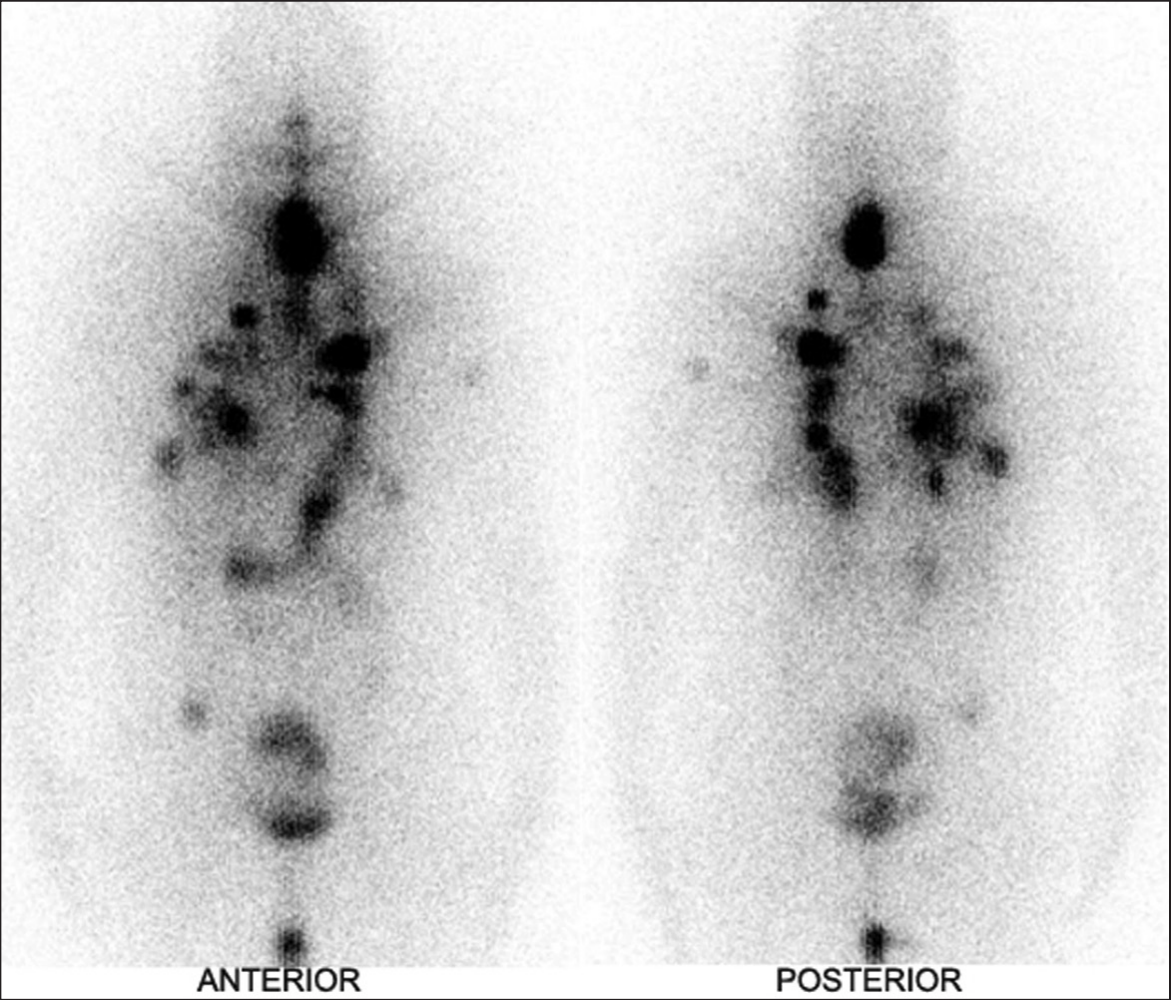
Post therapy whole body scan shows extensive I-131 avid metastases in both lungs

Hybrid SPECT-CT fusion imaging was then performed on the same camera. The focus in the neck was localized to the residual thyroid tissue, 1 cm below the lower border of the thyroid cartilage, on the left side [Figure 2a]. In addition, multiple radio-opaque nodules were detected in both the lungs, on the CT image. Radioiodine concentration was seen in a few of these nodules on the fused SPECT-CT images; most of the nodules did not show any radioiodine avidity at the dose used [Figure 2b‐d]. The patient was subsequently given high-dose radioiodine therapy at another institute by one of the authors (CSB). The post-therapy I-131 WBS acquired there showed extensive I-131 avid metastases in both lungs [Figure 3].

## DISCUSSION

The lung is one of the most common sites for distant metastases from differentiated thyroid carcinoma (DTC).[1] The reported rate of pulmonary metastases from DTC varies from 2 to 20%.[2] I-131 WBS plays an important diagnostic role in the detection of lung metastasis from DTC. However, in cases with considerable residual thyroid tissue, even post-therapy WBS may fail to show I-131 concentration in the lungs owing to significant neck uptake.[3] It has been reported that the sensitivity of a chest X-ray in the detection of lung metastases (52%) is lower than that of WBS (64%) and thoracic CT (82%).[4] It is generally believed that the CT images provided by a SPECT-CT system are of little diagnostic value as they do not possess the image properties of a diagnostic CT.[5] However, while the hybrid system CT component cannot replace a CT scan of diagnostic quality, it appears to be sufficient for the anatomical localization of scintigraphic foci.[6] Integrated I-131 SPECT-CT imaging has an additional value in patients with thyroid cancer, for characterization of equivocal tracer uptake seen on planar imaging as well as for precise localization of malignant lesions in the neck, chest, and skeleton. This localization of I-131 uptake may have a clinical impact on patient management by influencing referral for I-131 treatment, tailoring of the administered radioiodine dose, and / or the addition of surgery or external radiation therapy when indicated.[7] While most previous studies have performed this procedure after a therapeutic dose of radioiodine,[8] the present case illustrates that hybrid SPECT-CT fusion may identify the full extent of pulmonary metastases, when combined with low-dose (diagnostic) I-131 whole body scan.
